# Do all women shy away from competition? Competitive preferences among Dutch and non-Western females in the Netherlands

**DOI:** 10.1371/journal.pone.0321256

**Published:** 2025-05-27

**Authors:** Ozge Gokdemir, Devrim Dumludag, Ozlem Yorulmaz

**Affiliations:** 1 Faculty of Economics, Istanbul University, Istanbul, Turkey; 2 MPE, SBE, Maastricht University, Maastrciht, the Netherlands; 3 Faculty of Economics, Marmara University, Istanbul, Turkey; University of Amsterdam, NETHERLANDS, KINGDOM OF THE

## Abstract

This study explores the willingness to compete among native Dutch individuals and non-Western migrants in the Netherlands, with a particular focus on first-generation non-Western migrant women. Drawing upon existing literature on competitiveness and career preferences, this research suggests that non-Western migrants, especially the first generation of non-Western migrant women, may exhibit different levels of competitiveness compared to the native Dutch population or native Dutch women. This aspect is crucial for understanding the challenges non-Western immigrant women encounter in the job market and broader societal contexts. Utilizing data from two existing experiments conducted within the publicly accessible LISS framework, our research findings reveal that first generation of non-Western women demonstrate a higher propensity for competition than their Dutch counterparts do. Notably, this pattern is not replicated among non-Western men participants in all generations. The results align with research on gender equality and competitiveness in the countries of origin. In nations like the Netherlands, where gender equality is relatively high, notable differences in competitive preferences between native women and men are observed. However, this disparity is less pronounced among first-generation non-Western migrant women from countries with lower levels of gender equality. Additionally, the study uncovers variations in competitive preferences between native Dutch and non-Western migrant women within the same societal setting. This research contributes to a deeper understanding of the complex interactions among ethnicity, competitiveness, and gender dynamics in Dutch society.

## Introduction

Research on individual differences in the willingness to compete have gained significant importance in economics. Key studies by Gneezy *et al*. [1], Niederle and Vesterlund [2], and Buser *et al*. [3], have consistently highlighted gender disparities in this area. These studies link competitive inclination to career choices, emphasizing how competition plays a critical role in securing high paying jobs and promotions [4–6]. The literature points out that woman often struggle for recognition and cooperation in men dominated workplaces. This struggle tends to push men towards competitive settings more often than women, undervaluing women’s performance and affecting their career expectations [7]. Gender disparities in these contexts are largely attributed to cultural and social norms [8,9]. In this paper, we explore whether gender disparities in the willingness to compete vary across different ethnic groups within society. Nicholls [10] recently suggested that cultural and social norms might not only influence gender but also create variations in competitive tendencies among diverse ethnic groups. This perspective suggests a parallel between the experiences of women and ethnic minorities, suggesting that both groups may encounter similar societal challenges. By examining this aspect, we aim to uncover whether these societal norms lead to distinct competition tendencies among various ethnic groups, thereby contributing to a more comprehensive understanding of competition dynamics in a multi-ethnic society. There are four main explanations for why women might be less inclined to compete, as detailed in Table 1. Studies by Niederle and Vesterlund [2] and Bertrand [11] suggest that women may have a natural aversion to competitive environments. Researches by Croson and Gneezy [12] and Falk *et al*. [13] suggest that women tend to be more risk-averse, which can deter them from entering competitive situations. Findings by Barber and Odean [14] and Kamas and Preston [15] highlight that women often have lower self-confidence, which might affect their willingness to compete. Berlin and Dargnies [16] showed that women in particular, react more strongly to the feedback they receive also Chen *et al*. [17] found that women are expressions in real interpersonal interactions. These explanations shed light on why women may demonstrate a lower inclination toward competition. Interestingly, these theories also apply to migrants, suggesting a significant overlap in the experiences of women and minority groups. For instance, Siddique and Vlassopoulos [18] found that ethnic minorities are 25% less likely to participate in competitions in groups where they are not part of the majority ethnic group, compared to situations where all competitors share the same ethnic background. Bonin *et al*. [19] showed that first-generation immigrants in Germany have a lower risk appetite than natives. Constant *et al*. [20] demonstrated that migrants exhibit lower confidence levels than natives, thereby hindering the integration process for immigrants. Cohen *et al*. [21] also suggest that students from ethnic backgrounds tend to be more sensitive to feedback (see Table 1 for summary).

**Table 1:** Indicators explaining the avoidance of competition

Explanations	Women	Minorities
Distaste of competition	Niederle & Vesterlund, 2007; Bertrand, 2011	Siddique & Vlassopoulos, 2017
Risk aversion	Croson & Gneezy, 2009; Falk *et al*., 2018	Bonin *et al*., 2012
Low confidence	Barber & Odean, 2001; Kamas & Preston, 2012	Constant *et al*., 2009
Sensitive to negative feedbacks	Berlin & Dargnies, 2016 ; Chen at al., 2018	Cohen *et al*., 1999

These explanations suggest a significant overlap in the experiences of women and minority groups. Both groups often find themselves at a disadvantage, lacking access to key economic, cultural, and social resources. This commonality is rooted in the fact that both women and minorities are part of broader disadvantaged populations. Disadvantaged groups typically include individuals who face restricted access to essential resources within society, influencing their attitudes and behaviours towards competition.

The desire of individuals to compete is defined as “self-selection" and is observed differently in disadvantaged groups. The avoidance of competition by highly capable individuals from disadvantaged groups can negatively impact economic welfare. In the literature it is debated whether this behavior is innate or shaped by society. Research shows that women avoid competition because of societal norms and traditional gender roles. For example, comparisons between matrilineal and patriarchal communities demonstrate that it is possible for women to become more competitive [8] Additionally, the lack of encouragement for women at the candidacy level limits their representation in politics. Therefore, measures to promote women’s participation in politics are necessary to address these disparities [22]. Standard economic theory assumes rational behavior in the labor market, where workers and employers make decisions based on personal attributes, constraints, and goals. However, in practice, individuals with similar capabilities often face unequal treatment, particularly women and ethnic minorities [23]. This discrepancy, commonly referred to as the “ethnic penalty,” results in labor market disparities that persist regardless of observable characteristics [24–26].

As previously mentioned, ethnic minorities and women are generally considered disadvantaged groups in the labor market, frequently earning lower incomes than ethnic majorities and men [27–29]. While most studies focus on either the ethnicity wage gap [28,30–33] or the gender wage gap [27] separately, some have explored the simultaneous existence of both. These studies emphasize the compounded negative impact of ethnicity and gender on the wages of ethnic minority women in developed countries [33–35]

As a result, ethnic minority women may experience a dual penalty stemming from both their gender and ethnicity [36,37]. This persistent inequality not only affects their current labor market outcomes but also creates barriers for individuals yet to enter the workforce.

In the context of gender equality and its impact on competitive behavior, the existing literature appears to be relatively limited. Particularly, studies from countries with high levels of gender equality have shown unexpectedly larger gender gaps in tournament participation [38,39]. This indicates that in gender-equal societies, when women have equal access to resources (such as education, employment opportunities, and healthcare, which enable them to participate fully in society), their preferences may diverge, with some placing greater value on survival and independence [39].

Moreover, in countries with advanced economies and high gender equality, this trend is even more evident, with greater gender differences in economic preferences that go beyond a simple willingness to compete [40]. These findings underscore the nature of gender dynamics in competitive contexts, highlighting the need for a broader exploration that considers both societal factors and individual differences.

Building on this understanding, it becomes clear that gender disparities in the willingness to compete are not uniform across or within countries. These variances can be attributed to evolving demographic patterns and the varying degrees of gender equality across different regions. Specifically, the observed differences may arise from individuals originating from countries with diverse levels of gender equality. Thus, it is imperative to broaden the scope of analysis on gender disparities to encompass not only comparisons between countries but also the examination of individuals from various cultural backgrounds within the same nation.

Our research focuses primarily on immigrants in the Netherlands, with particular attention to first-generation immigrants. The study focuses on the first generation migrants because numerous scholars have found that second-generation immigrants tend to be more integrated into Dutch society, which makes it difficult to analyze the impact of the gender gap index effect. They typically have a greater proficiency in the Dutch language, more native friends, and their opinions, attitudes, and behaviors align more closely with those of the native Dutch population [41,42].

In the Netherlands, individuals with migrant backgrounds face additional challenges, including higher rates of unemployment, a tendency to secure lower-skilled occupations, and generally lower income levels compared to native majority counterparts [43]. Currently, a concerted effort is underway to conduct extensive research and formulate policies aimed at promoting equity and diversity. This effort is rooted in the understanding that when disadvantaged groups avoid competition, it not only constrains their participation and chances of success but also hinders their progress in achieving promotions and accessing higher-paying positions [2].

It is important to note that before 2022, CBS (Centraal Bureau voor de Statistiek) classified immigrants as either “Western” or “Non-Western.” In this system, first-generation immigrants were individuals born abroad, while second-generation immigrants were those born in the Netherlands with at least one parent born abroad. However, this type of classification faced scientific and sociological criticism, as the distinction between Western and Non-Western was considered debatable and did not fully reflect immigrants’ self-identification. Taking these critiques into account, CBS adopted a new classification system in 2022. There are two aspects to this new approach: born in the Netherlands or abroad.For a detailed explanation of the previous classification method distinguishing “Western” and “Non-Western” immigrants, further information can be found on the CBS website. However, since this study and the LISS data are from 2017 and 2018, the analysis in this paper will follow the previous classification, using the terms “Non-Western” immigrants.

To understand disparities in gender equality, we rely on the Gender Gap Index (GGI)of the World Economic Forum, a tool, also employed in the research of Falk and Hermle [40] and Markowsky and Beblo [39]. The Global Gender Gap Index is an annual assessment evaluating gender equality across four dimensions: Economic participation and opportunity, educational attainment, health and survival, and political empowerment. Analyzing the GGI for non-Western countries, where many immigrants in the Netherlands originate from, reveals that these countries score lower in gender equality compared to the Netherlands. This discrepancy suggests that immigrants might bring different perspectives on gender roles and competition [44].

Furthermore, this study explores the competitive tendencies of non-Western immigrants compared to Dutch natives. The choice to focus on this comparison stems from the predominant focus in migration literature on interactions and disparities between non-Western immigrants and the native Dutch population.

Additionally, research on gender equality and competitiveness tends to concentrate on Western countries. This further emphasizes the importance of our study’s focus on non-Western immigrants [38,39].

In this context, the aim of this study is to comprehensively investigate the competitive inclination between native Dutch individuals and non-Western immigrants in the Netherlands. Specifically, the study focuses on how ethnic background and gender intersect to shape competitive behaviors, addressing both ethnic and gender-based disparities. Consequently, Western immigrants have been excluded from the analysis. By exploring these dynamics, this study seeks to fill a significant gap in the literature and contribute to a nuanced understanding of competition in a multi-ethnic society. Therefore, this study addresses the following research questions: First, To what extent do competitive inclinations differ between native Dutch individuals and first-generation non-Western migrants in the Netherlands? Second, How do these differences vary across gender, particularly among women?

Our research fills a significant gap in the literature by exploring how individuals from non-Western backgrounds influence individuals’ competitive inclinations and examining the combined effects of ethnic background and gender on competitive preferences. The “double penalty” faced by minority women in job markets and broader society is a critical area of our study [36,38,39].

Building on this foundation, our study analyzes data from two experiments conducted within the LISS (Longitudinal Internet Studies for the Social Sciences) framework. These experiments allow us to examine the nuances of competitive behavior among non-Western minority groups in the Netherlands. Particular attention is given to women and different generational groups. By including both incentivized and hypothetical measures of competitiveness, we aim to capture a comprehensive picture of competitive tendencies across these subgroups.

This empirical approach enables us to uncover critical variations in competitive willingness between native Dutch individuals and non-Western migrants, particularly across gender and generational lines. The findings aim to inform policies and initiatives that promote equity and inclusion within diverse populations.

The rest of this paper is organized as follows: The next section introduces the experiments, including incentivized and hypothetical measures of competitiveness and the descriptive data from the LISS panel utilized in our analysis. The results section presents the empirical findings, focusing on gender and ethnic subgroups within the LISS sample, with additional analysis for women across different generations. The discussion section provides an in-depth examination of the results, focusing on the intersectionality of ethnicity and gender and addressing potential measurement errors. Finally, the conclusion summarizes our findings and discusses their implications for future research and policy.

## Experiment and data

### The experiment

The LISS panel is a publicly accessible and active online data platform. All experiments and surveys conducted on the LISS platform are available for use by researchers. Participants were approached through traditional methods, including letters, followed by telephone calls and/or house visits, inviting them to join the panel. Individuals not part of the original sample are ineligible to participate in assembled studies, thereby eliminating the possibility of self-selection.

This longitudinal study plays a crucial role in offering insights into individual responses to various policy shifts and societal transformations. Managed by CentERdata, a reputable survey research institute based at Tilburg University, the LISS panel comprises a representative sample drawn from the population register maintained by Statistics Netherlands [45,46]. Panelists engage in a variety of surveys and experimental studies, receiving compensation through a base fee, with additional incentives for experimental participation. The panel is committed to integrity, ensuring that all experiments are conducted without any form of deception.

Table 2 presents the distributions and means of key socio-economic variables for the entire population of the Netherlands, the LISS core samples, and the samples in the assembled studies, including the hypothetical and incentivized experiments.

**Table 2:** Representativeness of the LISS panel

Categories	Netherlands	LISS-2017	LISS-2018	Hypothetical	Incentivized
Men, %	49.60	49.07	49.03	47.07	44.76
Women, %	50,40	50.97	50.97	52.93	55.24
Age	41,6	42.29	41.98	51.7	45,6
Income	2625	2970	3000	2850	3285
Size of household	2.2	2.65	2,6	2.56	2.78
Primary, %	9.7	18.64	18.35	8.05	5.38
Secondary, %	61.6	51.35	50.16	56.00	56.08
Tertiary, %	28.6	30.01	31.49	35.95	38.55

Both the LISS 2018 and LISS 2017 panels include participants under the age of 15, which are not typically found in the general population. However, this deviation is not observed in the hypothetical and incentivized experimental samples. The data are from 2017 and 2018, sourced from Statista, CBS, and CentERdata. An overview of the distributions of key socio-economic variables among participants in two experiments is included in Table 2. The table shows that distributions and means are very similar in the experiments and the LISS samples except for age. The representation of age is skewed because a minimum age is required for participation in the assembled studies on the panel. This becomes especially noticeable in the hypothetical experiment. However, overall, the table suggests that the LISS sample and the experimental samples are effective in representing the socio-economic characteristics of the general population.

In March 2017, Thomas Buser and Hessel Oosterbeek conducted an online experiment on the LISS panel, adhering to the experimental design established by Niederle and Vesterlund [3].

This experiment collected hypothetical data through a survey that included questions on competitiveness, risk preferences, and confidence from all panel members. Participants encountered a hypothetical task involving the addition of two-digit numbers (e.g., 43+82+11+94+68=?) without using a calculator. They were offered a choice between two payment options: a piece rate (earning 1 Euro for each correctly solved problem) or a competition (competing against three randomly selected panel participants, with the potential to earn 4 Euros per correctly solved problem if they outperformed their opponents). The total time allocated for the experiment was five minutes for each subject.

Participants’ confidence in their performance was measured with questions such as, ’How well do you think you would perform compared to 9 other randomly selected LISS panel members in the above task? And where do you estimate your rank within this group?’ in the hypothetical experiment and ’How do you think you scored compared to other participants?’ in the incentivized experiment. Additionally, a 10-point scale was used in the hypothetical experiment, while an 11-point scale was used in the incentivized experiment. Results from the hypothetical experiment were reversed for consistency with the incentivized experiment: a score of 10 indicated the highest performance, while a score of 1 or 0 indicated the lowest within the group. Risk attitudes were assessed with the question, ’How do you see yourself? Are you generally someone who is willing to take risks, or do you try to avoid risks?’ Participants expressed their willingness to take risks on a scale from 0 (not at all willing to take risks) to 10 (very willing to take risks) [47].

**Fig 1:**
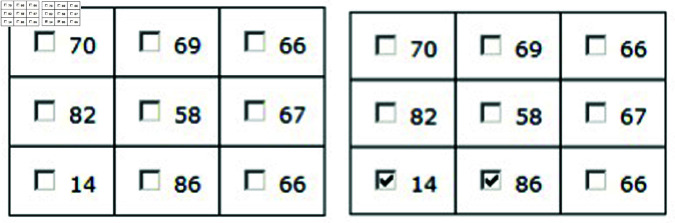
Example matrix task.

In April 2018, a specific subset of individuals from the LISS population was selected to take part in an incentivized choice experiment conducted by Thomas Buser. This experiment consisted of three rounds, each lasting two minutes where participants aimed to solve as many matrix problems as possible (as illustrated in Fig 1). The task involved selecting two numbers from a set of nine to find a combination that equaled 100.

In the beginning of the third round, participants were given a choice between two payment methods, similar to the hypothetical experiment: a “piece rate” or a “tournament” payment options. Under the piece rate option, participants would earn 40 cents for each correctly solved matrix. In contrast, the tournament option offered 80 cents for each correct solution, but only if the participant’s score surpassed that of a randomly selected peer; failing this, they would receive no payment.

Participants had the autonomy to select either the piece-rate and tournament schemes. In the tournament option, their scores would be compared to those of another participant, chosen randomly, regardless of the latter’s chosen payment method. The choice made in the third round, based on performance in the preceding two rounds, served as a measure of incentivized competitiveness. To assess participants’ confidence in the task, they were also asked to rate their performance on a scale from 0 (worst) to 10 (best) relative to other participants. This experiment also replicated the assessment of risk attitudes from the previous hypothetical scenario.

The two math tasks used in these experiments are real-effort tasks, widely used in experimental economics to study behavior that reflects real-world dynamics They measure behavior based on skill and effort rather than chance, providing insights into motivation and performance. By simulating competitive environments like the labor market, these tasks enable controlled and reliable analysis of willingness to compete.

The hypothetical experiment included a total of 4,596 respondents, while the incentivized experiment had 1,641 participants. Among these, 313 individuals in the hypothetical experiment and 163 in the incentivized experiment identified as having a non-Western ethnic background. Specifically, the hypothetical experiment included 195 first-generation non-Western participants, whereas the incentivized experiment included 100 first-generation non-Western participants.

The LISS dataset provides background variables related to origin, categorizing individuals based on CBS’ definitions (prior to 2022) into groups such as Dutch background, first-generation non-Western immigrants, and second-generation non-Western immigrants. However, these classifications are derived variables, meaning they are constructed by the dataset using underlying survey responses that are not directly visible to researchers.

Specifically, LISS determines these categories based on responses to questions such as: “Were you born in the Netherlands?,” “In what country were you born?,” “Was your father/mother born in the Netherlands?,” “In what country was your father/mother born?.” While researchers can only observe the final classification, the derivation process follows a predefined algorithm that utilizes detailed background information.

Before 2022, CBS classified first-generation non-Western immigrants as individuals born in a non-Western country with at least one parent also born in a non-Western country. Second-generation non-Western immigrants were those born in the Netherlands with at least one parent born in a non-Western country. This classification was based on predefined non-Western categories. However, in 2022, CBS replaced this system with a classification based on an individual’s country of birth and their parents’ country of birth. In this paper, participants were categorized into three groups: Dutch, first-generation non-Western, second-generation non-Western.

The choice to use both hypothetical and incentivized experiments in this study arises from the unique strengths and weaknesses of each method. Hypothetical experiments allow for large and diverse samples, improving the external validity of the findings. However, they are vulnerable to hypothetical bias, where participants may overestimate their willingness to compete without real monetary stakes [48,49]. Incentivized experiments reduce such biases by using real monetary rewards, leading to more accurate measures of willingness to compete [50]. However, their external validity is limited by smaller sample sizes and less geographic diversity due to higher costs and logistical challenges. By combining these methods, this study addresses the inherent limitations of each approach and offers a more comprehensive and robust analysis of willingness to compete.

### Descriptives

To investigate the relationship between our experimental measure of competitiveness and the demographic characteristics of native and immigrant populations, we utilized data from the LISS panel. This dataset includes information on income, education levels, and age. Given the skewed nature of income data, we applied logarithmic transformation for our analysis. This transformation revealed significant disparities between non-Western immigrants and natives; specifically, the log-transformed income for non-Western immigrants is markedly lower than that of natives, mirroring the income distribution in the Netherlands. Education levels were categorized into six groups, and age was segmented into seven groups, following the definitions provided by CBS (Statistics Netherlands).

Table 3 displays the distribution of relative frequencies among participants, categorized by ethnicity and gender (Dutch women and men, non-Western women and men), willingness to compete, and various demographic indicators such as education level and marital status.

**Table 3:** Distribution of relative frequencies of individuals from different gender and ethnicity participating in the experiments based on demographic indicators and ethnicity (%)

	Hypothetical				Incentivized			
	**Dutch**		**Nonwestern**		**Dutch**		**Nonwestern**	
	**Men**	**Women**	**Men**	**Women**	**Men**	**Women**	**Men**	**Women**
Marital status								
Married	60.50	54.53	44.87	61.15	57.71	56.28	44.59	40.45
Not married	39.50	45.47	55.13	38.85	42.29	43.72	55.41	59.55
Employment								
Respondent has not paid work	46.62	54.84	43.57	56.64	20.99	35.81	37.50	53.49
Respondent has a paid work	53.38	45.16	56.43	43.36	79.01	64.19	62.50	46.51
Competition								
Piece work	61.63	76.40	55.77	63.06	63.62	78.70	64.38	73.86
Competition	38.37	23.60	44.23	36.94	36.38	21.30	35.62	26.14
Age								
15 - 24 years	5.64	7.34	11.54	13.38	4.09	7.27	5.41	12.36
25 - 34 years	9.80	11.93	16.67	29.30	14.19	16.96	18.92	33.71
35 - 44 years	12.97	13.79	17.95	21.66	17.87	17.07	20.27	19.10
45 - 54 years	17.52	17.85	23.08	18.47	28.10	24.78	25.68	21.35
55 - 64 years	19.60	20.28	18.59	14.01	30.56	31.17	27.03	13.48
65 years and older	34.46	28.81	12.18	3.18	5.18	2.75	2.70	0
Education								
Primary school	6.10	6.94	13.73	10.90	3.69	3.41	8.33	11.24
Vmbo	18.59	25.65	15.69	16.67	16.53	18.50	19.44	16.85
Havo/Vwo	9.17	10.79	11.76	13.46	10.52	10.35	12.50	12.36
Mbo	26.18	22.64	24.18	26.28	27.73	29.07	27.78	25.84
Hbo	27.02	25.17	14.38	17.95	28.28	28.08	6.94	19.10
Wo	12.94	8.80	20.26	14.74	13.25	10.57	25	14.61

In the Dutch participant group, the proportion of married women and men is higher than that of their unmarried counterparts in both experiments. Conversely, among non-Western men, the proportion of unmarried individuals is greater than that of married individuals. In the hypothetical experiment, similar to Dutch women, married women have a higher proportion, whereas the situation is reversed in the incentivized experiment.

In the incentivized experiment, a higher proportion of women and men among Dutch participants possess jobs compared to those without. This trend is similarly observed among non-Western men. However, non-Western women have a higher proportion of individuals without jobs. Regarding the hypothetical experiment, men with jobs are more prevalent compared to those without, and women without jobs outnumber those with jobs.

Overall, individuals opting for piece work without engaging in competition are proportionally more represented in both experiments, independent of gender. Notably, this disparity is more pronounced among women compared to men.

When examining age distribution, it is evident that among Dutch participants, the 55+ age group is more prominent compared to other age groups, regardless of gender. In contrast, there appears to be a concentration among non-Western participants in the 35–54 age range. Interestingly, the youngest participant group consists of non-Western women.

Among Dutch participants, HBO and MBO levels represent the highest educational attainment, followed by VMBO. However, for non-Western men, the highest proportion is found in MBO-WO, while among women, it is solely in MBO. Another striking point is that the share of primary school participants among non-Western individuals is higher in both experiments compared to their Dutch counterparts.

An examination of the differences in extroversion and intellect among Dutch women and non-Western women, as well as Dutch men and non-Western men, revealed no statistically significant differences (*p*>0.10). However, Dutch women showed higher average scores in conscientiousness compared to their non-Western counterparts, and Dutch men also exhibited higher average scores than non-Western men in the hypothetical experiment (*p*<0.01). In the incentivized experiment, this difference was statistically significant only among women ethnic groups (*p*<0.01).

Regarding stability, both Dutch women and men attained significantly higher average scores than their non-Western peers in both experimental conditions. In terms of agreeableness, Dutch women recorded significantly higher average scores than non-Western women across both experiments (*p*<0.01), while no statistically significant differences were observed among men based on ethnicity (*p*>0.10).

In terms of confidence levels, it is observed that Dutch women have significantly higher average scores compared to their non-Western counterparts only in the incentivized experiment (*p*<0.01). For men, no statistically significant differences are found between ethnic groups in either experiment (*p*>0.01). Regarding risk-taking, a higher average score is observed among non-Western men compared to Dutch men only in the hypothetical experiment (*p*<0.01). For women, the differences between ethnic groups are not statistically significant in either experiment (*p*>0.01).

## Results

Table 5 presents probit regression results for various subgroups within the LISS sample, including the entire sample, as well as women and men participants.The results are presented separately for both the hypothetical and incentivized experiments, analyzing the relationship between the dependent variable “willing to compete” and various independent variables.

**Table 4:** Descriptive statistics for Dutch and Non-Western groups according to gender subsamples

	Hypothetical						Incentivized					
	**Women**			**Men**			**Women**			**Men**		
	**Dutch**	**Non-Western**	**Diff**	**Dutch**	**Non-Western**	**Diff**	**Dutch**	**Non-Western**	**Diff**	**Dutch**	**Non-Western**	**Diff**
Risk taking	5.005	5.165	–.161	5.608	6.121	–.514***	4.507	4.414	.093	5.337	5.444	–.107
	(1.982)	(2.369)	[.166]	(2.014)	(2.007)	[.167]	(1.924)	(2.567)	[.223]	(2.007)	(2.135)	[.249]
Confidence	5.249	5.159	.090	5.481	5.379	.101	4.623	4.081	.542***	5.258	5.388	–.130
	(1.861)	(2.021)	[.154]	(2.120)	(2.364)	[.176]	(1.759)	(2.081)	[.201]	(1.751)	(2.038)	[.219]
Score of round 1	–	–	–	–	–	–	9.379	8.338	1.042**	10.840	8.689	2.151***
							(3.975)	(4.226)	[.444]	(4.475)	(5.031)	[.552]
Score of round 2	–	–	–	–	–	–	8.356	6.911	1.446**	9.237	7.852	1.389**
							(5.634)	(5.722)	[.626]	(5.951)	(5.444)	[.720]
Extroversion	3.231	3.164	.067	3.234	3.240	–.006	3.216	3.124	.092	3.238	3.250	–.012
	(.662)	(.654)	[.060]	(.675)	(.676)	[.059]	(.683)	(.629)	[.078]	(.700)	(.686)	[.086]
Conscientiousness	3.799	3.617	.182***	3.733	3.602	.131***	3.836	3.575	.261***	3.712	3.609	.102
	(.505)	(.526)	[.046]	(.520)	(.539)	[.046]	(.507)	(.518)	[.058]	(.528)	(.548)	[.065]
Stability	3.397	3.123	.275***	3.648	3.466	.182***	3.351	3.108	.243***	3.649	3.409	.239***
	(.701)	(.648)	[.063]	(.682)	(.672)	[.060]	(.717)	(.641)	[.082]	(.714)	(.674)	[.087]
Intellect	3.448	3.429	.019	3.535	3.453	.083	3.474	3.400	.074	3.603	3.500	.103
	(.507)	(.489)	[.046]	(.509)	(.493)	[.045]	(.517)	(.484)	[.059]	(.507)	(.497)	[.063]
Agreeable	4.035	3.783	.251***	3.700	3.725	–.026	4.039	3.745	.294***	3.676	3.682	–.005
	(.457)	(.529)	[.042]	(.518)	(.547)	[.046]	(.464)	(.521)	[.054]	(.538)	(.568)	[.067]

( ) = standard deviation, [ ] = standard error, *** =p≺0.01

**Table 5:** Probit results for all, women and men

	All sample				Women				Men			
	**Hypothetical**		**Incentivized**		**Hypothetical**		**Incentivized**		**Hypothetical**		**Incentivized**	
Dependent variable: competition												
Variables	*Est*.*Coef*.	*SE*	*Est*.*Coef*	*SE*	*Est*.*Coef*.	*SE*	*Est*.*Coef*	*SE*	*Est*.*Coef*.	*SE*	*Est*.*Coef*	*SE*
Non-Western migrants	0.188**	0.094	0.282**	0.139	0.435***	0.136	0.444**	0.206	–0.092	0.134	0.096	0.205
*Dutch (ref)*												
Risk	0.151***	0.012	0.213***	0.023	0.147***	0.018	0.200***	0.031	0.159***	0.017	0.235***	0.035
Confidence	0.048***	0.111	0.102***	0.027	0.014	0.018	0.081**	0.037	0.075***	0.015	0.141***	0.043
Education	0.025	0.017	0.140***	0.032	0.004	0.025	0.137***	0.047	0.042*	0.025	0.149***	0.046
Age	–0.024	0.020	–0.028	0.035	–0.009	0.027	–0.005	0.049	–0.040	0.030	–0.035	0.052
Gender	–0.202***	0.050	–0.126	0.088		–	–	–	–	–	–	–
Household size	0.038	0.023	0.012	0.036	0.043	0.033	0.009	0.050	0.027	0.034	0.014	0.052
Employment	0.062	0.054	0.099	0.097	0.105	0.078	0.018	0.129	0.042	0.079	0.266*	0.155
Marital status	0.017	0.055	–0.222**	0.094	0.054	0.078	–0.263**	0.132	–0.015	0.079	–0.191	0.136
Log income	0.050	0.052	0.189**	0.092	–0.005	0.071	0.282**	0.123	0.107	0.080	0.024	0.143
Round 1 score	–	–	0.025**	0.011	–	–	0.018	0.011	–	–	0.028*	0.015
Round 2 score	–	–	0.011	0.008	–	–	0.019*	0.011	–	–	0.005	0.011
*Personality traits*												
Extroversion	0.087**	0.040	–0.123*	0.034	0.109*	0.058	–0.121	0.092	0.071	0.055	–0.121	0.090
Intellect	0.100*	0.054	0.123	0.087	0.102	0.076	0.107	0.119	0.083	0.078	0.112	0.130
Conscientiousness	0.044	0.050	–0.115	0.083	0.077	0.072	–0.118	0.118	0.030	0.070	–0.108	0.118
Agreeable	–0.164***	0.053	–0.013	0.009	–0.262	0.082	–0.000	0.013	–0.068	0.070	–0.023*	0.012
Stability	0.017	0.036	0.063	0.059	–0.024	0.052	–0.013	0.086	0.050	0.051	0.084*	0.084
Observations	3612		1465		1874		801		1738		664	

*Note:* ***p ≺ 0.01, **p ≺ 0.05, *p ≺ 0.10.

We explore the correlation between choosing the competitive payment scheme and different ethnic backgrounds. As the incentivized experiment is conditional on performance, we have incorporated a control variable, the average score from previous rounds. In these regressions, Dutch participants, as well as specific subsets of Dutch women and Dutch men, are used as the reference categories for ethnicity.

In the hypothetical and incentivized experiments, non-Western immigrants are significantly more likely to be willing to compete than Dutch participants. Interestingly, within the women subgroup, non-Western women also exhibit a strong preference for competition, a trend not observed among men.

Risk and confidence factors consistently exhibit positive and significant relationships with the willingness to compete across nearly all subgroups. It’s noteworthy that the confidence variable specifically reveals a positive association with the willingness to compete among women, particularly in incentivized experiments. However, confidence is not statistically significant (p≻0.10) for women in hypothetical experiment.

Other variables such as education, age, gender, household size, employment, marital status, personality traits, round scores and log transformed income demonstrate varying effects across different conditions and subgroups. Education is positively associated with competition, particularly in incentivized experiments, for both the entire sample and men. However, the relationship is weaker and sometimes insignificant for women, indicating potential gender disparities in how education influences competitive behavior. Being married negatively affects willingness to compete, particularly in incentivized experiments for women, suggesting that societal or familial roles may influence women’s competitive behavior. Higher income positively influences competition, especially for women in incentivized experiments, possibly reflecting greater financial security enabling risk taking. The results also show that personality traits influence willingness to compete differently. Extroversion encourages competition in hypothetical experiment but not with incentives, while agreeableness consistently reduces it, especially for women. Intellect shows a slight positive effect in theory but not in practice, and emotional stability has minimal impact, mainly for men in incentivized experiments. Conscientiousness appears unrelated to willingness to compete.

The results from Table 5 concerning women highlight the need for a more thorough examination. Therefore, we have utilized the generational specification made available by the LISS data. The LISS data offers insights into the generational status of Western and non-Western immigrants in the Netherlands, categorizing those born abroad as the first generation and those born in the Netherlands as the second generations.

Table 6 presents probit regression results for women from different generational backgrounds in both hypothetical and incentivized experiments. The generational categories include the first generation of non-Western women, second generation of Non-Western women with Dutch women serving as the reference category.

**Table 6:** Probit regression results for women from different generations

	Hypothetical		Incentivized	
**Variables**	**Est. Coef.**	**SE**	**Est. Coef**	**SE**
First gen. of Non-Western women	0.626***	0.171	0.716***	0.248
Second gen. of Non-Western women	0.143	0.210	0.119	0.278
*Dutch women (ref)*				
Risk	0.148***	0.018	0.204***	0.032
Confidence	0.013	0.018	0.078**	0.037
Education	0.006	0.025	0.142**	0.047
Age	–0.012	0.027	–0.013	0.050
Household size	0.040	0.033	0.001	0.050
Employment	0.102	0.078	0.017	0.129
Marital status	0.047	0.078	–0.266**	0.132
Log income	0.003	0.071	0.309**	0.125
Round 1 score	–	–	0.019	0.016
Round 2 score	–	–	0.019*	0.011
*Personality traits*				
Extroversion	0.110*	0.058	–0.122	0.092
Intellect	0.102	0.076	0.107	0.119
Conscientiousness	0.077	0.071	–0.122	0.118
Agreeable	–0.260***	0.082	–0.000	0.013
Stability	–0.025	0.052	–0.013	0.086
Observations	1874		801	

*Note:* ***p ≺ 0.01, **p ≺ 0.05, *p ≺ 0.10.

The results indicate that first-generation Non-Western women display a significantly positive coefficient in both hypothetical and incentivized experiments, implying a greater willingness to compete in comparison to Dutch women. However, there are no significant findings for the second generation of Non-Western women. The risk variable has a positive and statistically significant impact on willingness to compete in both hypothetical and incentivized experiments. On the other hand the variable “confidence” does not yield a statistically significant impact on willingness to compete in either of the experiments. Education is significant in incentivized experiments but not in hypothetical experiment. This suggests that education may have a greater impact on competitive behavior when real rewards are involved. Being married is significant and negative in incentivized experiments, indicating that married women are less likely to compete when real stakes are involved. This might again reflect societal or familial responsibilities that discourage married women from engaging in competitive scenarios with financial consequences. Moreover women with higher incomes are significantly more likely to compete, potentially due to greater financial security. Among personality traits, agreeableness significantly reduces willingness to compete in hypothetical scenarios, while extroversion shows a weak positive effect. These findings highlight generational differences and the role of contextual factors like incentives and personality in shaping competitive behavior among women.

## Discussion

### Gender equality perspective

In the context of gender equality and its impact on willingness to compete, numerous studies have highlighted the existence of gender disparities [38,39].

Interestingly, research conducted in countries with higher gender equality, as measured by GGI, has revealed larger gender gaps in tournament entry [38,39]. These findings raise intriguing questions about the influence of gender equality in shaping competitive behavior. In non-Western countries, where patriarchal values and traditional gender roles are more prevalent, the dynamics of competition might significantly differ.

The results of our probit regression analysis shed light on these dynamics, particularly among the first-generation non-Western immigrants. Specifically first-generation non-Western women, who were born abroad, display a significantly higher willingness to compete compared to Dutch women in both the hypothetical and incentivized experiments. This observation suggests that the focus on meeting basic needs and ensuring independence, which may be more pronounced in non-Western cultures, could influence the competitive behavior of these women. This emphasizes the importance of cultural and social factors in understanding competitiveness.

To further investigate this phenomenon, we adopted an alternative perspective. If women in countries with higher gender equality are less willing to compete than men, it can be expected that in countries with lower gender equality the competitive behaviors of women and men might be more aligned, driven by women’s emphasis on meeting basic needs and achieving independence. This scenario could result in a negligible gender difference in competitiveness. For this reason, we conducted a robustness analysis focusing on the first generation of non-Western immigrants and Dutch individuals. The purpose of this analysis was to ascertain whether gender differences in competitive behavior manifest differently across societies with varying levels of gender equality.

Table 7 presents the results of a proportion test examining the descriptive statistics of competition between men and women for both the Dutch and the first generation of non-Western participants. Among the Dutch participants, men consistently exhibit a significantly higher willingness to compete than women across both experimental conditions. In contrast, no significant gender differences are observed among first-generation non-Western participants in either experimental condition, indicating a consistent pattern in this group.

**Table 7:** Descriptive statistics for Dutch and non-Westerns

	Hypothetical			Incentivized		
Groups	Men	Women	Diff	Men	Women	Diff
Dutch	.388	.236	–.157***	.364	.213	–.151***
	(.487)	(.425)	[.014]	(.481)	(.409)	[.022]
First generation Non-Westerns	.410	.400	–.010	.341	.245	–.096
	(.494)	(.492)	[.071]	(.479)	(.434)	[.923]
Second generation Non-Westerns	.483	.265	.218***	.379	.286	.094
	(.041)	(.033)	[.052]	(.090)	(.076)	[.118]

( ) = standard deviation, [ ] = standard error, ***p≺0.01.

For second-generation non-Western participants, the results are less consistent. A significant gender difference in competition rates is found in the hypothetical experiment (*p*<0.001), but not in the incentivized experiment (*p*>0.05). This inconsistency may reflect the cultural duality experienced by second-generation participants, who are potentially influenced by both Dutch and non-Western cultural norms. Unlike the Dutch and first-generation participants, second-generation individuals display varying competitive behaviors depending on the experimental context, which may suggest a transitional stage in their cultural integration.

Additionally, our findings indicate that first-generation non-Western women in our sample show no lesser willingness to compete than their male counterparts. This aligns with results reported by Dariel *et al*. [51], which found no significant gender differences in willingness to compete in the United Arab Emirates.

These findings highlight the challenges of studying gender differences in competitiveness within migration contexts. Dutch and first-generation non-Western participants show consistent patterns in both experiments, but the mixed results for second-generation participants suggest a need to better understand how cultural integration and dual influences affect competitiveness.

Lastly, while prior research often focuses on inter-country comparisons based on country-specific factors, this study provides valuable insights into differences within a single country, particularly in the context of migration. The results highlight the influence of cultural and social factors on individuals’ willingness to compete and show the importance of considering diverse contextual factors when analyzing gender disparities in competitiveness.

**Fig 2:**
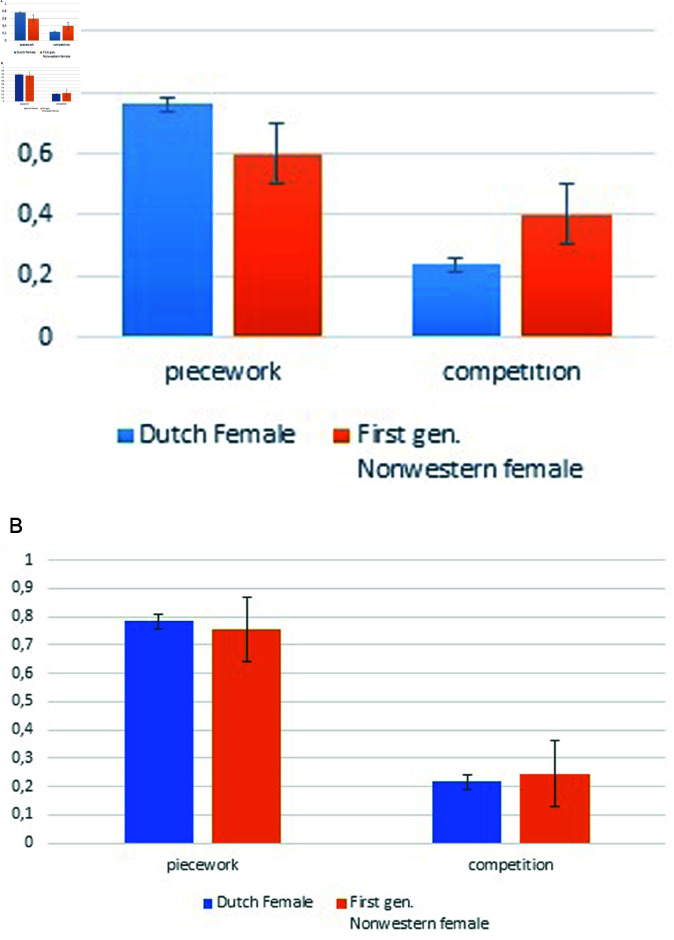
Comparison of willingness to compete. ( **a**) Hypohetical experiment; ( **b**) incentivized experiment.

As shown in Fig 2a and 2b, we can see differences in willingness to compete between Dutch women and first-generation non-Western women, with the difference being more pronounced in the hypothetical experiment than in the incentivized experiment.

Moreover, these observations indicate that competitive preferences not only vary across countries but also within them.This implies that immigrants from different backgrounds may exhibit distinct behavioral patterns, potentially influenced by the context of gender equality in their countries of origin.

### Examining possible measurement errors

In 2019, Gillen, Snowberg, and Yariv made a significant contribution to the discourse in experimental economics by questioning whether competitiveness should be regarded as an independent behavior or a composite measure influenced by risk preferences or confidence [52]. In contrast, a 2021 study by Buser, Niederle, and Oosterbeek provided compelling evidence suggesting that competitiveness, risk, and confidence are distinct constructs that yield different outcomes [53].

Given these academic discussions, we conducted ordered probit regressions to investigate the reasons behind the increased levels of competitiveness exhibited by first-generation non-Western women compared to Dutch women. This heightened competitiveness might be attributed to a greater propensity for risk-taking or overconfidence among non-Western women.

The emphasis on the first generation of non-Western women is underpinned by literature suggesting a correlation between risk propensity and migration patterns. Jaeger *et al*. [54] suggest that people who are more willing to take risks are also more likely to migrate. It is generally acknowledged that the decision to migrate is predominantly made by the first generation of migrants. Consequently, if this generation exhibits a tendency towards risk-taking, such behavior can be detected through regression analysis, which may highlight potential measurement errors.

An additional aspect we sought to investigate, highlighted in Table 4, was the noticeable difference in risk-taking tendencies between non-Western and native Dutch participants during the hypothetical experiment. Subsequent analyses aimed to differentiate these differences across genders through separate regression and analyses.

Moreover Kamas and Preston [55] found out that confidence, as measured by expected ranking, is the primary factor influencing decisions to participate in tournaments. Consequently, we employed expected ranking as a measure of confidence, reflecting an individual’s assessment of their own ability or performance relative to others. If the first generation of non-Western women are more confident in their correctness and rank themselves higher relative to Dutch women, it may highlight potential measurement errors as well.

Therefore, risk behavior and confidence were separately used as independent variables in the ordered probit regressions to provide insights into the underlying factors shaping the observed disparities. If the first generation of non-Western migrant women are statistically significantly more inclined towards risk-taking and exhibit greater confidence compared to Dutch women, this could indicate measurement errors.

In Table 8, two ordered probit regression results are presented. The results show that, compared to Dutch women, being a part of the first generation of non-Western female group does not result in statistically significant differences in risk-taking behavior and confidence.

**Table 8:** Ordered Probit results for women

	Hypothetical		Incentivized		Hypothetical		Incentivized	
Dependent variable: Risk					Dependent variable: Confidence			
Variables	*Est*.*Coef*.	*SE*	*Est*.*Coef*	*SE*	*Est*.*Coef*.	*SE*	*Est*.*Coef*	*SE*
First gen. of Non-Western women	0.153	0.136	–0.160	0.177	–0.125	0.136	–0.111	0.180
Second gen. of Non-Western women	0.246	0.158	0.181	0.195	–0.420***	0.157	–0.290	0.198
Dutch women (ref)								
Observations	1874		801		1917		801	

***p ≺ 0.01, **p ≺ 0.05, *p ≺ 0.10

Controls:age,hholdsize,education,maritalstatus,logincome,employment,big5 (round scores added for incentivized)

In the regression where the confidence was the dependent variable, a statistically significant negative association was observed between belonging to the non-Western female group and confidence in the hypothetical experiment. However significant relationship was not observed in the incentivized experiment.

Table 9 presents the t-test results analyzing the descriptive statistics for both Dutch women and first generation of Non-Western women. Assessing women in both the hypothetical and incentivized experiments revealed no significant effect of ethnicity on risk-taking and confidence is. This suggests that the average levels of risk-taking for Dutch and Non-Western women are comparable.

**Table 9:** Descriptive statistics of risk behavior and confidence levels for Dutch Women and First Generation of Non-Western Women

	Hypothetical			Incentivized		
Variable	Dutch women	First Gen. Women NW	Diff	Dutch women	First Gen. Women NW	Diff
Risk Behavior	5.001	5.105	–.093	4.512	4.250	.262
	(1.994)	(2.358)	[.210]	(1.964)	(2.392)	[.283]
Confidence	5.24	5.41	–.173	4.60	4.21	.383
	(1.863)	(2.076)	[.196]	(1.787)	(1.923)	[.256]

Consequently, it becomes evident that the competitiveness of non-Western women cannot be fully explained by differences in risk-taking behavior and confidence when compared to Dutch women.

## Conclusion

This study introduced a comprehensive investigation into how ethnicity, gender, and competitiveness interact in the Netherlands. It revealed significant insights, particularly a pattern of higher competitiveness among non-Western migrant women, especially the first generation, compared to native Dutch individuals. This pattern not only underlines the role of gender in shaping competitive preferences but also highlights how cultural and socio-economic backgrounds influence these behaviors.

Importantly, our research contributes to the broader discourse on gender disparities in competitive settings, challenging the assumption that gender equality straightforwardly translates into similar competitive inclinations across genders. Instead, the results underscore a paradox within societies known for high gender equality, like the Netherlands, where larger gender gaps in competitiveness are observed, particularly among natives. For non-Western migrant women, their willingness to compete may reflect underlying motivations tied to survival and independence, attributes potentially sharpened by the socio-economic and cultural contexts of their countries of origin.

The absence of a statistically significant gender gap in willingness to compete among the first generation of non-Western migrants, as opposed to the significant gap found within the Dutch sample, suggests a reevaluation of how gender equality impacts competitive behaviors across different cultural contexts. This observation inspires a reconsideration of the conventional wisdom regarding the relationship between gender equality and competitive inclination, highlighting the need for a more differentiated approach that takes into account cultural and societal nuances.

In conclusion, our study addresses a significant gap in existing literature by exploring how ethnic background and gender affect competitive preferences. It provides valuable insights for policymakers, educators, and organizations working to create an inclusive environment that accommodates the diverse competitive inclinations of individuals from various backgrounds. By recognizing and addressing the unique challenges faced by non-Western migrant women, and all individuals, in competitive situations, it is possible to make significant progress toward achieving equity and inclusion in the workforce and society. The experimental findings provide valuable insights into competitive behavior therefore future studies should explore real-world settings, focus on different groups, and combine experiments with long-term field data to better understand competitiveness.

## Declaration of generative AI and AI-assisted technologies in the writing process

During the preparation of this work the authors used ChatGPT4/OPENAI for language editing. After using this tool/service, the authors reviewed and edited the content as needed and take full responsibility for the content of the publication.
